# Early recognition of tomato gray leaf spot disease based on MobileNetv2-YOLOv3 model

**DOI:** 10.1186/s13007-020-00624-2

**Published:** 2020-06-08

**Authors:** Jun Liu, Xuewei Wang

**Affiliations:** Facility Horticulture Laboratory of Universities in Shandong, Weifang University of Science and Technology, Weifang, Shandong 262700 China

**Keywords:** YOLOv3, Convolutional neural network, Tomato diseases and pests, Object detection

## Abstract

**Background:**

Tomato gray leaf spot is a worldwide disease, especially in warm and humid areas. The continuous expansion of greenhouse tomato cultivation area and the frequent introduction of foreign varieties in recent years have increased the severity of the epidemic hazards of this disease in some tomato planting bases annually. This disease is a newly developed one. Thus, farmers generally lack prevention and control experience and measures in production; the disease is often misdiagnosed or not prevented and controlled timely; this condition results in tomato production reduction or crop failure, which causes severe economic losses to farmers. Therefore, tomato gray leaf spot disease should be identified in the early stage, which will be important in avoiding or reducing the economic loss caused by the disease. The advent of the era of big data has facilitated the use of machine learning method in disease identification. Therefore, deep learning method is proposed to realise the early recognition of tomato gray leaf spot. Tomato growers need to develop the app of image detection mobile terminal of tomato gray leaf spot disease to realise real-time detection of this disease.

**Results:**

This study proposes an early recognition method of tomato leaf spot based on MobileNetv2-YOLOv3 model to achieve a good balance between the accuracy and real-time detection of tomato gray leaf spot. This method improves the accuracy of the regression box of tomato gray leaf spot recognition by introducing the GIoU bounding box regression loss function. A MobileNetv2-YOLOv3 lightweight network model, which uses MobileNetv2 as the backbone network of the model, is proposed to facilitate the migration to the mobile terminal. The pre-training method combining mixup training and transfer learning is used to improve the generalisation ability of the model. The images captured under four different conditions are statistically analysed. The recognition effect of the models is evaluated by the F1 score and the AP value, and the experiment is compared with Faster-RCNN and SSD models. Experimental results show that the recognition effect of the proposed model is significantly improved. In the test dataset of images captured under the background of sufficient light without leaf shelter, the F1 score and AP value are 94.13% and 92.53%, and the average IOU value is 89.92%. In all the test sets, the F1 score and AP value are 93.24% and 91.32%, and the average IOU value is 86.98%. The object detection speed can reach 246 frames/s on GPU, the extrapolation speed for a single 416 × 416 picture is 16.9 ms, the detection speed on CPU can reach 22 frames/s, the extrapolation speed is 80.9 ms and the memory occupied by the model is 28 MB.

**Conclusions:**

The proposed recognition method has the advantages of low memory consumption, high recognition accuracy and fast recognition speed. This method is a new solution for the early prediction of tomato leaf spot and a new idea for the intelligent diagnosis of tomato leaf spot.

## Background

Tomato is an important economic crop in the world. It is easily affected by many kinds of diseases, and this condition severely affects the quality and yield of tomato and causes huge economic losses. Tomato gray leaf spot is a common disease in tomato planting; this disease not only damages the leaves but also destroys the photosynthesis of the leaves, affects the growth of tomato and reduces the yield. In recent years, tomato gray leaf spot has been a severe outbreak in domestic tomato production base. The tomato gray leaf spot disease is difficult to control. The infection process of tomato gray leaf spot pathogen can be divided into four stages: contact, invasion, latency and onset periods. Contact period refers to the period during which pathogens contact with host plants; invasion period refers to the period during which pathogens invade the host and establish a parasitic relationship with it; latency period refers to the period during which pathogens start to show obvious symptoms from establishing a parasitic relationship with the host, during which pathogens absorb nutrients, spread and propagate in the host, and the symptom is mild; once the appropriate environment is encountered, the disease enters the onset stage and spread rapidly, and the symptom becomes severe. Therefore, if early detection of tomato gray leaf spot disease can be achieved before the large-scale epidemic, then prevention and control programmes can be formulated as early as possible. Appropriate prevention and control measures should be taken, and passive prevention and control should be changed to active prevention and control in advance. The prevention and control effect will be greatly improved, and the loss will also be minimised. Early detection of diseases can also reduce the use of pesticides and environmental pollution and ensure tomato quality safety and human health. Therefore, the early recognition of tomato gray leaf spot is an excellent way to inhibit the rapid development of the disease and even avoid the disease. Traditional methods of disease detection cannot meet the needs of large-scale planting, and the plants often miss the best control period because of low diagnosis efficiency and rapid spread of disease [1, 2]. The application of image processing technology in crop disease detection at home and abroad has achieved good results. Image processing technology can quickly and accurately distinguish the types of diseases according to the characteristics of diseases. In this way, the disease prevention strategies can be adopted timely and measures can be taken to prevent further expansion of diseases.

In the past, people used to judge the class of tomato disease subjectively through experience, but the ability to distinguish amongst multiple diseases is limited and the process is time consuming.

The machine learning image processing technology is developing rapidly and is widely used in all aspects, including the agricultural field. Applying machine learning and image processing technology to crop disease recognition has incomparable advantages over traditional manual diagnosis and recognition methods [3]. People only need to collect a small number of disease image samples. The process involves the following steps: firstly, the dataset is pre-processed; secondly, the feature extraction algorithm is used to extract the features of the disease area in the image; lastly, the obtained feature information is sent to the classifier for training and the model parameters are obtained. The generated model can be used to detect the disease category. Training with a large number of datasets is time consuming due to the lack of datasets and the poor generalisation ability of the model. Moreover, the development of agricultural modernisation towards the direction of intelligence has highlighted the shortcomings of these traditional image detection methods.

The development of new technology has enabled not only discovering the characteristics of things artificially but also collecting a large number of data, designing algorithms and programming, mining laws from data and building models by using advanced computer hardware facilities. Deep learning is a representative branch of artificial intelligence. Although this concept was only introduced in 2012, various network models have been produced after its development [4–10]. At present, deep learning is widely used in various fields, especially in computer vision. It efficiently solves the tasks of image classification, object detection and semantic segmentation. Compared with the traditional pattern detection method, the disease detection method based on the deep convolutional network (CNN) abandons the sophisticated image pre-processing and feature extraction operations and uses the end-to-end structure to simplify the detection process. CNN can be used to train the model prediction results, which not only can save time and workforce but also can make a real-time judgment, as long as a large number of crop disease image datasets can be obtained. This way greatly reduces the great losses caused by the disease.

In recent years, object detection based on deep learning has rapidly developed. Researchers have proposed increasingly sophisticated network structure to improve the accuracy of object detection, including RCNN [11] (Region-Convolutional Neural Network), SPP-Net [12] (Spatial Pyramid Pooling-Networks) and Faster-RCNN [13]. These object detection networks can achieve high accuracy but can only achieve a frame rate below 1 frame/s due to the limited computing power and memory resources of the embedded platform.

In the aspect of deep neural networks for mobile object detection, researchers have proposed some miniaturised deep neural networks. For example, SSD [14] (Single Shot MultiBox Detector) and YOLO [15] (You Only Look Once) have been introduced to improve the detection speed. They use high-performance GPU to achieve the effect of real-time object detection. However, the speed of object detection will decrease obviously when these models are applied to the embedded platform. The reason is that the performance of the GPU of the embedded platform is far less than that of PC, and the performance of the former is at least 1/10 lower than that of the latter. At present, some scholars are improving and designing lightweight convolutional models, such as MobileNet [16] and SqueezeNet [17], according to the application requirements of mobile and embedded devices. However, their practical application is rarely reported.

In the study of plant diseases, the traditional machine learning technology has a good application effect, and deep learning is a major step to promote this study. Its powerful learning ability improves the performance and precision of neural networks. It is a recently popular technology for visual image analysis. The application of deep learning technology in plant disease recognition has become a major research task in this field. Current research on plant diseases and insect pests based on deep learning involves various crops, including different vegetables, fruits and food crops. The tasks completed include classification and detection of diseases and insect pests. At present, few public datasets on plant diseases and pests are available. Researchers usually find the best solution by comparing different training and test dataset proportions and network models. However, gaps exist in complexity between these susceptible images and real field scenarios. Solving the problem of real-time field pest detection based on mobile devices can still be developed.

This study aims to achieve a proper balance between the accuracy of object detection and real-time performance (that is, reduce the size of the model whilst ensuring the computing power of the embedded platform to meet the computing demand of the model). Thus, this study introduces a small network architecture with small computing power demand and stable object detection effect, that is, MobileNetv2-YOLOv3, on the basis of the latest research results of CNN theory and the characteristics of the tomato grey leaf spot image. The proposed network improves the detection speed whilst improving the detection accuracy and ensures the detection accuracy of the model whilst minimising the volume of the network model.

## Related Work

### Existing image recognition methods of plant disease identification

Traditional plant disease identification method based on computer vision technology usually needs to extract the texture, shape, vein, colour and other features of the disease spots. This method has low recognition efficiency because it depends on rich expert knowledge in the field of agricultural diseases. With the rapid development of artificial intelligence technology in recent years, many researchers have conducted relevant research based on deep learning technology to improve the accuracy of plant disease identification. The existing analysis methods of plant diseases are mainly disease classification.

Mohanty et al. [18] used GoogleNet and AlexNet to classify and recognise 54,306 diseased and healthy plant leaf images in the PlantVillage dataset and draw a conclusion that the average classification effect of GoogleNet is slightly better than that of AlexNet. The accuracy of the trained deep convolutional neural network model on the test set is up to 99.35%. The method of training deep learning model on a growing and publicly available image dataset is a clear way to identify plant diseases in horticultural crops assisted by intelligent mobile phones. Amara et al. [19] identified disease types of 60 × 60 coloured banana leaves based on LeNet. Deep learning also plays a major role in the detection of plant disease severity. Wang et al. [20] trained a series of deep convolutional neural networks to diagnose the severity of diseases by using the apple black rot images in the PlantVillage dataset. The performances of shallow networks trained from scratch and deep models tuned by transfer learning were also evaluated. The best model is deep VGG16 trained by transfer learning, and the overall accuracy in the test set is 90.4%. Ferentinos et al. [21] used an open database containing 87,848 images to identify 58 kinds of diseases of 25 different plants based on deep learning, and the best performance reaches 99.53% in terms of accuracy rate. Barbedo (2019) [22] studied plant disease identification from individual lesions and spots using the GoogLeNet architecture, and the obtained accuracy ranges from 75% to 100% for each crop. This variation is caused by differences in the number of images, the number of diseases, conditions and the levels of difficulty.

Convolutional neural networks (CNNs) usually require a large number of samples for training. However, collecting training data required by models is difficult and costly in many applications [23]. Therefore, the research on data expansion is particularly important. In previous studies, many researchers have combined deep learning with transfer learning under the condition of limited datasets [24], and a tool for plant disease recognition and classification has been developed using image processing unit (GPU). Srdjan [25] proposed an evaluation method of deep learning model to identify 14 different classes of plant diseases, including 13 classes of disease and healthy plant leaf images. The dataset size is 30,880 images and the average accuracy reaches 96.3% for this method combined with the transfer learning method. Liu et al. [26] enhanced the training dataset by rotating, mirroring and adding Gaussian noise, brightness adjustment and contrast adjustment. This method increases the size of the training set by 12 times and reduces the over-fitting problem.

In addition to the expansion of data volume, improvements in deep-learning algorithms are critical to the disease recognition results. Too et al. [27] researched on a deep network architecture and used images from the PlantVillage dataset to form data sizes of 34,727 training set samples, 8702 validation set samples and 10,876 test set samples. The comparative test verified that DenseNets needs fewer parameters and reasonable calculation time to achieve the most advanced performance compared with VGG and ResNet. The test accuracy achieves 99.75%. Picon et al. [28] adopted an improved algorithm based on deep residual neural network to deal with the detection of various plant diseases under actual acquisition conditions, amongst which different adaptations for early disease detection have been proposed. The results obtained showed that the AuC index of all analysed diseases is higher than 0.80. Selvaraj et al. [29] retrained three different CNN architectures using the transfer learning method. By using pre-trained disease recognition models, deep transfer learning was performed to generate networks that could make accurate predictions. Zhong et al. [30] proposed three methods of regression, multilabel classification and focus loss function based on DenseNet-121 CNN to identify apple leaf diseases. The proposed methods achieve 93.51, 93.31 and 93.71% accuracy on the test set.

The disease recognition methods in the above-mentioned research are different from disease detection, which cannot automatically locate the disease area from the image and needs to extract the disease area manually for recognition. Deep learning can also be applied to plant disease identification. However, the current research in this field is still at an early stage, especially in practical application, due to the continuous improvement of the requirements for plant disease and pest identification under sophisticated background, such as high recognition accuracy, short calculation time, improved system robustness and strong generalisation ability.

### Research progress of tomato disease image recognition

In tomato disease image classification, Durmus et al. [31] classified and recognised 10 kinds of tomato diseases in the PlantVillage dataset by using AlexNet and SqueezeNet model. The experiment found that the classification accuracy of AlexNet is slightly higher than that of SqueezeNet, but the size of the model and the time taken are doubled. Brahimi et al. [32] found that the performance of the CNN is better than that of the shallow convolutional network, and the performance of the model can be improved by initialising the model parameters with pre-training weights. On this basis, nine kinds of tomato diseases are identified. By fine-tuning the AlexNet and GoogLeNet model, the accuracy reaches 99.18%. Aravind et al. [33] used AlexNet and VGG16 combined with transfer learning to identify seven kinds of tomato diseases; the experiment showed that the accuracies are 97.29 and 97.49%. Although transfer learning can make the model converge quickly and achieve better recognition effect, it is limited by the original network structure. The original AlexNet and VGG16 models have a sophisticated structure and many parameters, which greatly limit the practical application and deployment of the model. Karthik et al. [34] proposed an attention-based deep residual network to detect the infection type of tomato leaves. The experiment used the PlantVillage dataset, amongst which 95,999 images were used as training models and 24,001 images were used for validation. The dataset included three diseases, namely, early blight, late blight and leaf mold. The experimental results showed that the proposed attention-based residual network can utilise the features of CNN learning at various processing levels and achieves 98% overall accuracy on the validation set in five-fold cross-validation.

In tomato disease object detection, Fuentes et al. (2017) [35] proposed a method based on deep learning to detect diseases and pests of tomato plant images captured by various resolution camera devices. Three kinds of object detectors of CNN were used, and they were called ‘deep learning meta-architectures’. These meta-architectures were combined with ‘deep feature extractor’ to show the performance of deep meta-architecture and feature extractor. The method of local and global class annotation and data expansion was used to improve the accuracy of training and reduce the number of false-positives. The end-to-end training and testing were conducted on the large-scale tomato disease dataset. The experimental results showed that the system can effectively identify nine different types of pests and diseases and can deal with sophisticated scenes from the surrounding areas of plants. Fuentes et al. (2018)  [36] proposed an improved algorithm of tomato disease and insect pest detection aiming at the problem of false alarm and classification imbalance of tomato diseases and insect pests. The framework is mainly composed of three units: (1) main diagnosis unit (bounding box generator), which generates bounding box, and the bounding box contains the region and category of diseases and insect pests; (2) auxiliary diagnosis unit (CNN filter bank), which trains each independent CNN to filter the samples of error classification; (3) integration unit, which combines the information of independent diagnosis unit and auxiliary diagnosis unit whilst maintaining the true positive samples to eliminate the false positives. The experiments showed that the recognition rate of this method is approximately 96%. Fuentes et al. (2019) [37] proposed a method that not only can effectively detect and locate plant anomalies but also can produce diagnostic results, display abnormal locations and describe sentence symptoms as output. In the newly established dataset of tomato plant anomaly description, the average accuracy is 92.5%. Zhang et al. [38] proposed an improved Faster-RCNN method to detect healthy tomato leaves and four diseases. Firstly, the deep residual network was used instead of VGG16 for image feature extraction to obtain deep disease features. Secondly, the boundary boxes were clustered using a k-means clustering algorithm. The anchor was set on the basis of the clustering results. Lastly, k-means experiments were performed on three different feature extraction networks. The experimental results showed that the improved detection method for crop leaf diseases achieves 2.71% higher recognition accuracy than the original Faster-RCNN method. However, in the literature, Faster-RCNN was used to detect objects, which needs to be done in two steps: the region recommendation was extracted firstly and then detected. The prevailing YOLO can directly generate coordinates and probabilities for each category through expressions. Therefore, the real-time performance of existing research needs to be improved.

### Existing problems and development trend of current research

1. Previous research has only focused on the application of the deep neural convolutional network of each variety to coarse-grained disease identification and ignored the early detection of the diseases. In the actual production, the early and late-stage images of the same disease have different characteristics. If the disease is identified accurately in the early stage of disease occurrence and corresponding control measures are taken, the loss caused by the disease can be greatly reduced. However, the location of early stage disease is relatively hidden, and the area of infection is also small, which occupies a low proportion of pixels in the whole image. Therefore, the problem of using the CNN to extract early tomato disease features and identify fine-grained disease remains to be solved.
2. Different areas of tomato diseases show different image characteristics: some of them are flaky, whereas others have a punctate pattern. The characteristics of the same disease will change in different stages; some gradually progress from point to pieces. In the early stage of the disease, the lesion is relatively small and occupies a small area in the whole image. Therefore, tomato diseases areas have the characteristics of irregular and small size, which make the object detection process difficult. For the images of tomato diseases collected in the real natural environment, the background part (such as weeds and ground) is similar to the tomato disease area to a certain extent, and the existing object detection algorithm will cause a lot of false detection, which will result in reduced accuracy.
3. The speed of real-time computing on the mobile terminal is difficult to achieve, which is unsuitable for scenes with strong real-time requirements, such as intelligent mobile phones.
4. In the previous research, influencing factors on the accuracy of deep learning models applied to plant pathology are rarely involved. Barbedo, J.G.A. (2018) [39] argued that many factors may affect the accuracy of deep learning models applied to plant pathology. The robustness of the proposed model to different kinds of conditions that are commonly found in practice should be determined.

In view of the above-mentioned problems, this study proposes a real-time detection method for tomato gray leaf spot under sophisticated background. This method can effectively extract the early stage of tomato gray leaf spot characteristics, train and test the images of tomato gray leaf spot and achieve the purpose of real-time and accurate positioning of tomato gray leaf spot area. Thus, the proposed method can provide a technical support for the mobile-oriented intelligent diagnosis system of tomato leaf spot disease.

## Materials and methods

### Dataset used in the research

The experience and suggestions of agricultural experts indicate that the pathogen of tomato gray leaf spot takes about 24 h to propagate from a large number to invade the host under suitable meteorological conditions. If the meteorological conditions remain within the range suitable for the growth and breeding of the pathogen after the invasion and the duration is close to or greater than the incubation period of the pathogen, then the tomato gray leaf spot will further develop and various lesions may appear in about 3 days. Therefore, early detection in this study means that the symptoms of tomato gray leaf spot can be considered early up to 3 days after the initial infection. The dataset must be images of the disease in its early stage in a real environment to realise the early detection of tomato grey leaf spot images. The gathering place of tomato gray leaf spot images is the Shouguang tomato planting base in Shandong Province in China. The images are captured using a variety of equipment, such as a digital camera and a smart mobile phone, under natural light; 2166 copies of original images are collected, including cloudy, sunny and rainy days, to cover all lighting conditions. To ensure the diversity of tomato gray leaf spot image dataset, 219 tomato gray leaf spot images are obtained through a web crawler, and the number of images in the dataset is 2385.

The aforementioned dataset is annotated using the LabelImg tool. Considering the corresponding relationship between labels and data and ensuring uniform distribution of the dataset, the dataset is randomly divided into training, verification and test datasets according to the proportion of 70, 10 and 20% by Matlab. The final dataset is stored in the format of the PASCAL VOC dataset. In accordance with the diagnostic criteria and recommendations of agricultural experts, the test dataset is divided into four parts: sufficient light (sunny days) without leaf shelter, sufficient light (sunny days) with leaf shelter, insufficient light (cloudy days) without leaf shelter and insufficient light (cloudy days) with leaf shelter. The final dataset is shown in Table 1.

**Table 1:** Datasets and size

Dataset	Training set	Validation set	Test set	Total number
Number of images	1669	477	239	2385
Number of annotated samples	9847	2814	1410	14,071

### Principle of MobileNetv2-YOLOv3 model

#### Principle of YOLOv3 model

The Yolo algorithm was proposed by Redmon et al. [15] in 2016. The object detection task in this algorithm is transformed into a regression problem, which greatly accelerates the detection speed. YOLOv3 [40] is proposed based on YOLOv2 [41], the detection speed of YOLOv2 is maintained and the detection accuracy is greatly improved. YOLOv3 uses the idea of the residual neural network [42]. The introduction of multiple residual network modules and the use of multiscale prediction improve the shortcomings of YOLOv2 network in small object recognition. This algorithm is one of the best algorithms in object detection because of the high accuracy and timeliness of its detection. This model uses several 3 × 3 and 1 × 1 convolution layers with good performance, and some residual network structures are also used in the subsequent multiscale prediction. This model has 53 convolution layers and can also be called Darknet-53.

The loss function of the object detection network of YOLOv3 is shown in Formula (1).

1 $$ \begin{aligned} \\&Loss\, = \,Loss_{coord} \, + \,Loss_{obj} \,\, + Loss_{class} \hfill \\& = \,\lambda_{coord} \,\sum\limits_{i = 0}^{{s^{2} }} {\sum\limits_{j = 0}^{B} {l_{ij}^{obj} \,\left[ {\left( {x_{i} - \,\mathop {x_{i} }\limits^{{ \wedge }} } \right)^{2} \, + \,\left( {y_{i} - \,\mathop {y_{i} }\limits^{{ \wedge }} } \right)^{2} \,} \right]} } \hfill \\& + \,\lambda_{coord} \,\sum\limits_{i = 0}^{{s^{2} }} {\sum\limits_{j = 0}^{B} {l_{ij}^{obj} \,\,\left[ {\left( {\sqrt {w_{i} } \, - \sqrt {\mathop {w_{i} }\limits^{{ \wedge }} } } \right)^{2} \, + \,\left( {\sqrt {h_{i} } \, - \sqrt {\mathop {h_{i} }\limits^{{ \wedge }} } } \right)^{2} \,} \right]} } \hfill \\& \quad + \sum\limits_{i = 0}^{{s^{2} }} {\sum\limits_{j = 0}^{B} {l_{ij}^{obj} \,} \,\left( {C_{i} \, - \,\mathop {C_{i} }\limits^{{ \wedge }} } \right)}^{2} \, + \lambda_{noobj} \sum\limits_{i = 0}^{{s^{2} }} {\sum\limits_{j = 0}^{B} {l_{ij}^{obj} \,} \,\left( {C_{i} \, - \,\mathop {C_{i} }\limits^{{ \wedge }} } \right)}^{2} \hfill \\& \,\quad + \,\mathop \sum \limits_{i = 0}^{{S^{2} }} l_{ij}^{obj} \,\sum\limits_{c \in class} {\left( {p_{i} \,\left( c \right)\, - \,\mathop p\limits^{{ \wedge }}_{i} \left( c \right)} \right)}^{2} \hfill \\& \quad \hfill \\ &\end{aligned} $$.

In the above-mentioned formula, $i$ represents the $i$ square, $j$ represents the $j$ bounding box predicted by the square, $obj$ indicates the existence of an object, $noobj$ indicates the absence of an object, $C_{i}$ is the class of the predicted object, $\mathop C\limits^{{ \wedge }}_{i}$ is the class of the real object and $\lambda_{coord}$ and $\lambda_{noobj}$ are the penalty coefficients. The loss function consists of five parts. The first part contains the predicted and actual $x$, $y$ coordinates, and this part is used to calculate the loss of the predicted central coordinate. The second part contains the predicted and actual width and height, namely, $w$ and $h$, and this part is used to calculate the loss of width and height of the predicted bounding box. The third part comprises the bounding box confidence loss, and the regression goal is the IoU value of the predicted and actual bounding boxes. The first three parts only calculate the loss when a corresponding relationship exists between the predicted and actual bounding boxes. The fourth part is the loss of confidence without corresponding objects. The third and fourth parts are the loss of confidence in the prediction process. The fifth part is the loss of classification. The loss of classification is calculated only when objects exist in the bounding box.

#### Bounding regression loss function based on GIoU

**Fig. 1:**
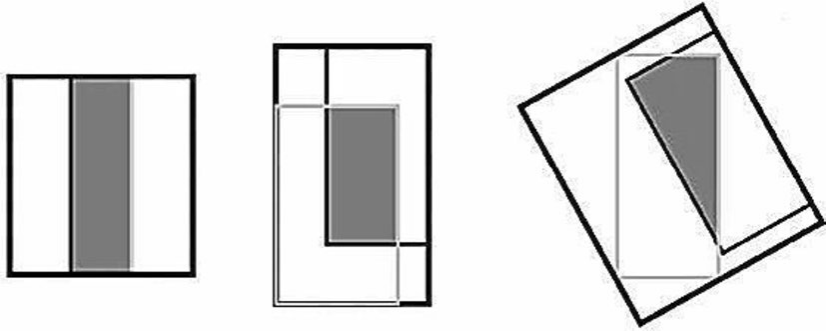
Diagram of two overlapping rectangles. The black rectangle represents the predicted bounding box, and the gray rectangle represents the original marked bounding box

IoU is the degree of coincidence between the prediction and marked bounding boxes in the original image. The bounding box regression IoU value is often used as the evaluation index in object detection. However, most detection frameworks do not combine this value to optimise the loss function. IoU can be back propagated and optimised directly as an objective function. Considering the choice between the optimisation measure and the use of alternative loss functions, the best choice is to optimise the measure. Traditional IoU as a loss function has two disadvantages: if the two objects do not overlap, then the IoU value will be 0, and the gradient will be 0, which cannot be optimised; if two objects overlap in different directions and the intersection level is the same, then their IoU will be exactly the same. IoU cannot accurately reflect the degree of coincidence between two objects. Figure 1 shows that using three different methods to overlap two rectangles can achieve the same IoU value, but their coincidence degree differs. The regression effect of the leftmost graph is the best, and the regression effect of the rightmost graph is the worst. The prediction bounding box of the rightmost graph is the rotation candidate bounding box [43]. Therefore, the value of the IoU function does not reflect the overlap of the two objects. In the detection of tomato gray leaf spot, the accuracy of regression box directly determines the success rate of detection. Therefore, the shortcomings of IoU are solved by introducing GIoU. IoU value range is [0,1], whilst GIoU has a symmetric interval and a value range of [− 1,1]; the maximum value of 1 is taken when the two coincide, and the minimum value of −1 is taken when the two do not intersect and are infinitely far away. Consequently, GIoU is a good distance measurement index. Unlike IoU, which only focuses on overlapping areas, GIoU focuses not only on overlapping areas but also on other non-overlapping areas, which can better reflect the coincidence degree of the two. GIoU loss can replace the loss function of bounding box regression in most object detection algorithms, as shown in Formulas (2)–(5).

2 $$ IoU = \frac{{\left| {\left( {A \cap B} \right)} \right|}}{{\left| {\left( {A \cup B} \right)} \right|}} $$

3 $$ GIoU = IoU - \frac{{\left| {C\left( {A \cap B} \right)} \right|}}{\left| C \right|} $$

4 $$ L_{GIoU} = 1 - GIoU $$

5 $$ \begin{aligned} GIoULoss\, = \,&\lambda_{coord} \,\sum\limits_{i = 0}^{{S^{2} }} {\sum\limits_{j = 0}^{B} {l_{ij}^{obj} } } \,\left( {1\, - \,GIoU} \right)\, + \,\sum\limits_{i = 0}^{{S^{2} }} {\sum\limits_{j = 0}^{B} {l_{ij}^{obj} } } \,\left( {C_{i} \, - \mathop C\limits^{{ \wedge }}_{i} } \right)^{2} \hfill \\ \quad \quad \quad \quad + \;&\lambda_{noobj} \,\sum\limits_{i = 0}^{{S^{2} }} {\sum\limits_{j = 0}^{B} {l_{ij}^{obj} } } \,\left( {C_{i} \, - \mathop C\limits^{{ \wedge }}_{i} } \right)^{2} \hfill \\ \quad \quad \quad \quad + \,&\sum\limits_{i = 0}^{{S^{2} }} {l_{ij}^{obj} } \sum\limits_{c \in class} {\left( {p_{i} \,\left( c \right)\, - \,\mathop p\limits^{{ \wedge }}_{i} \,\left( c \right)} \right)}^{2} . \hfill \\ \end{aligned} $$

In the above formula, $A$ and $B$ are any two rectangular boxes, $C$ is the smallest circumscribed rectangle surrounding $A$ and $B$ and $S$ is the space of $A$ and $B$.

#### Network design of MobileNetv2-YOLOv3

Traditional YOLOv3 uses the self-defined backbone network Darknet-53, the model calculation is sophisticated, and the storage space requirements are high. The calculation speed of a 416 × 416 image on GPU is 30 ms, and the calculation speed on CPU is 255.8 ms. This study proposes a lightweight neural network model for real-time object detection called MobileNetv2-YOLOv3 network, which is designed on the basis of traditional YOLOv3. The inferential speed of GPU is 16.9 ms, and the inferential speed of CPU is 80.9 ms. MobileNet [16] is a lightweight neural network based on mobile terminal. This study uses MobileNetv2 [44] as the backbone network of MobileNetv2-YOLOv3. The proposed model combines the anti-residual module with the depth-wise separable convolution. Firstly, the number of channels is increased by 1 × 1 convolution; secondly, the depth-wise convolution is performed by 3 × 3 convolution; lastly, the dimension is reduced by 1 × 1 convolution.

This study changes the feature image fusion to make deep connection at 19 and 34 layers to avoid reducing the object detection accuracy of small objects by using MobileNetv2 network. For the input image of 416 × 416, 13 × 13 feature image is obtained by convolution after the convolution network at 53 layers. The feature image receptive field here is the largest, which is suitable for the detection of large objects, that is, the first prediction output. To achieve fine-grained object detection, the 53-layer feature map of convolution layer starts to up sample from the right and obtains the feature map of the same resolution with 34 layers. After the residual module is fused with the 34-layer feature map, the 65 layers obtain the feature map of 26 × 26 after convolution, which has medium-sized receptive field and is suitable for detecting medium-sized objects. The 65-layer feature map is up sampled again and obtains the feature map of the same resolution with 19 layers. The feature map is fused with the 19-layer feature image through the residual module. The 52 × 52 feature image is obtained by eight times lower sampling than the input image. At this time, the receptive field is small, which is suitable for detecting small-sized objects.

The proposed MobileNetv2-YOLOv3 is an end-to-end object detection framework based on the idea of regression. The use of depth-wise separable convolution to extract features can effectively improve the computational efficiency of the convolutional network and reduce its huge number of parameters. At the same time, the detection accuracy of the convolutional network model is improved using multilayer feature fusion and point convolution to increase the network depth.

In the channel dimension mapping of the feature map, the conventional convolution is assumed be transformed and decomposed into linear combinations. If $K$ represents a regular convolution kernel, then

6 $$ K = M \cdot {\bigwedge }\left( b \right). $$

In the above-mentioned formula, $b$ is an $m$-dimensional matrix composed of two-dimensional convolution of $S \times S$ size, which can be expressed as

7 $$ b = \left[ {\begin{array}{*{20}c} {b_{00}^{i} } & \cdots & {b_{0s}^{i} } \\ \vdots & \ddots & \vdots \\ {b_{s0}^{i} } & \cdots & {b_{ss}^{i} } \\ \end{array} } \right]. $$

${\bigwedge }\left( b \right)$ is defined as a diagonal matrix, and the diagonal elements are $\,b_{i\,} \,\left( {i\, = \,0,\,1,\,2, \cdots ,\,m} \right)$, which can be expressed as

8 $$ {\bigwedge }\left( b \right) = \left[ {\begin{array}{*{20}c} {b_{0}   } & 0 & \cdots & 0 \\ 0 & {b_{1} } & \cdots & 0 \\ \vdots & \vdots & \vdots & \vdots \\ 0 & 0 & \cdots & {b_{m} } \\ \end{array} } \right]. $$

$M$ is defined as the numerical matrix of $n \times m$ $n$ and $m$ represent the dimensions of the output and input feature maps, respectively. “$\cdot$” represents a special matrix multiplication. The operation formula of conventional convolution kernel $K$ can be expressed as

9 $$ \begin{aligned} K = \left[ {\begin{array}{*{20}c} {k_{00} } & \cdots & {k_{0m} } \\ \vdots & \ddots &\vdots \\ {k_{n0} } &\cdots &{k_{nm} } \\ \end{array} } \right] = \left[ {\begin{array}{*{20}c} {\mu_{00} b_{0} } &\cdots & {\mu_{0m} b_{m} } \\ \vdots &\ddots &\vdots \\ {\mu_{n0} b_{0} } &\cdots &{\mu_{nm} b_{m} } \\ \end{array} } \right]\, \hfill \\ \quad = \left[ {\begin{array}{*{20}c}& {\mu_{00} } &\cdots &{\mu_{0m} } \\ \vdots &\ddots &\vdots \\ {\mu_{n0} } &\cdots &{\mu_{nm} } \\ \end{array} } \right] \cdot \left[ {\begin{array}{*{20}c} {b_{0} } &\cdots &0 \\ \vdots &\vdots &\vdots \\ 0 &\cdots &{b_{m} } \\ \end{array} } \right]. \hfill \\ \end{aligned} $$

In the aforementioned formula, $\mu_{nm}$ is the weight coefficient of convolution kernel on position $\left( {n,\,\,m} \right)$. $k_{nm}$ is the value of convolution kernel on position $\left( {n,\,m} \right)$, which can be expressed as $k_{nm} = \mu_{nm} b_{m}$.

According to the above-mentioned formula, the number of parameters of conventional convolution is $s \times s \times n \times m$. The number of depth-wise separable convolution parameters of MobileNetv2-YOLOv3 model that meets the requirements of mobile terminal is $s \times s \times m + n \times m$. The compression rate is

10 $$ \frac{s \times s \times m + n \times m}{s \times s \times n \times m} = \frac{1}{n} + \frac{1}{s \times s}. $$

**Fig. 2:**
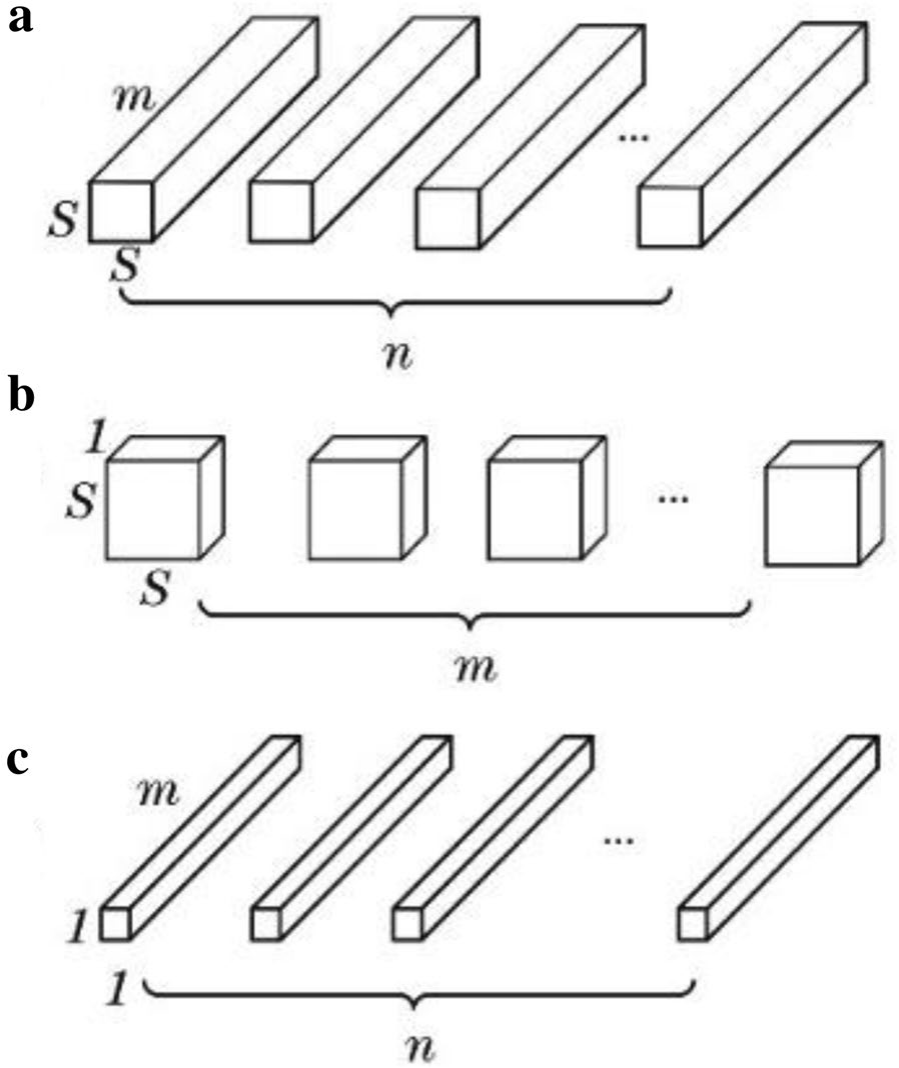
Basic structure of depth-wise separable convolutional network **a** standard convolution filter; **b** depth-wise separable convolution filter; **c** point convolution filter

The batch normalisation (BN) method is used to activate the corresponding operation. The mean value of the output dimensions is set to 0, and the variance is set to 1. This setting can reduce the change in the input data distribution of the next layer network and unify the distribution of the input data. It can also effectively improve the model convergence speed and avoid gradient explosion. The basic structure of the depth-wise separable convolutional network is shown in Fig. 2. The figure shows that the conventional convolution kernel is replaced by two convolution nuclei, that is, depth-wise separable and point convolutions are adopted.

The MobileNetv2-YOLOv3 model is constructed using the depth-wise separable convolution mentioned above. The network is a full convolution network, which consists of regular, depth-wise separable and point convolutions. For the output of each convolutional network, BN is adopted, that is, the BN layer is added. The use of the BN layer solves the gradient disappearance and explosion in the process of back propagation.

According to Huang et al. [45], the feature map can be obtained from the shallow layer network and can be fused to obtain better performance. YOLOv2 [41] only conducts monolayer feature map fusion. In the proposed model, a new feature map fusion method called multilayer feature map fusion is adopted. The method changes the channel of feature map by point convolution method, uses reshape method to transform the feature map to the specified size, undergo the same process for the multilayer network, overlay with the upper network, and obtains the fused feature maps. A deep and narrow network architecture is designed on the basis of SSD [14]. The deeper network architecture can obtain higher precision. By contrast, the narrower network architecture limits the complexity of the network. Therefore, the proposed MobileNetv2-YOLOv3 model has fewer network layers than other object detection networks. Point and depth-wise separable convolutions are used to construct the network to limit its complexity. In the main convolution operation at the end of the network structure, the depth of the network is increased by increasing the number of point convolution to limit the complexity of the network.

#### Pre-trained method combining mixup training and transfer learning

Various types of tomato gray leaf spot exist in the natural environment, and mutual shielding of tomato leaves is often encountered due to the limited scenarios covered by the sample dataset. Thus, the detection of tomato disease will limit the generalisation ability of the model. In this study, the visual coherent image mixup method designed for training object detection network is used. The method is effective in enhancing the general ability of the model. Mixup refers to combining two input images into one image according to a certain weight. The training model based on this composite image is robust and can effectively reduce the effect of the differences between images.

Transfer learning transfers the knowledge learned from the trained model to the new model to help the training of the new model. Yosinski et al. [46] conducted transfer learning experiment. They proved that the underlying CNN could learn general features of objects, such as geometric, edge and colour changes. By contrast, the high-level network is responsible for extracting specific feature details. A small dataset can also achieve good training effect through transfer learning.

In this study, a hybrid training method is adopted on COCO dataset for preliminary training, and the knowledge learned from COCO dataset is transferred to tomato gray leaf spot image recognition through transfer learning. By freezing part of the convolution layer, only correct model parameters of part of the convolution layer for back propagation are obtained. By using a method combining mixup training and transfer learning, the training time can be reduced, the memory consumption can be saved and tomato gray leaf spot object recognition effect can be improved.

### Metrics used to evaluate the proposed method

In this study, F1 score and AP (average precision) are used to evaluate the model trained by the loss function. The formula is expressed as follows.

11 $$ P = \frac{TP}{TP + FP}. $$

12 $$ R = \frac{TP}{TP + FN}. $$

13 $$ F1 = \frac{2PR}{P + R}. $$

14 $$ AP = \mathop \smallint \limits_{0}^{1} P\left( R \right)dR. $$

In the above-mentioned formula, P is the accuracy rate, R is the recall rate, TP is the number of true positive samples, FP is the number of false positive samples and FN is the number of false negative samples.

### Experimental operation environment

The experimental hardware environment of this study is shown in Table 2. On this basis, the software environment is built as follows: Ubuntu 16.04, python, OPENCV and CUDA. The framework uses the Caffe and Darknet-53 frameworks.

**Table 2:** Configuration of experimental hardware environment

Hardware name	Model	Number
Main board	Asus WS X299 SAGE	1
CPU	INTEL I7-9800X	1
Memory	The Kingston 16G DDR4	2
Graphic card	GEFORCE GTX1080Ti	2
Solid-state drives	Kingston 256G	1
Hard disk	Western digital 1T	1

### Model training

**Fig. 3:**
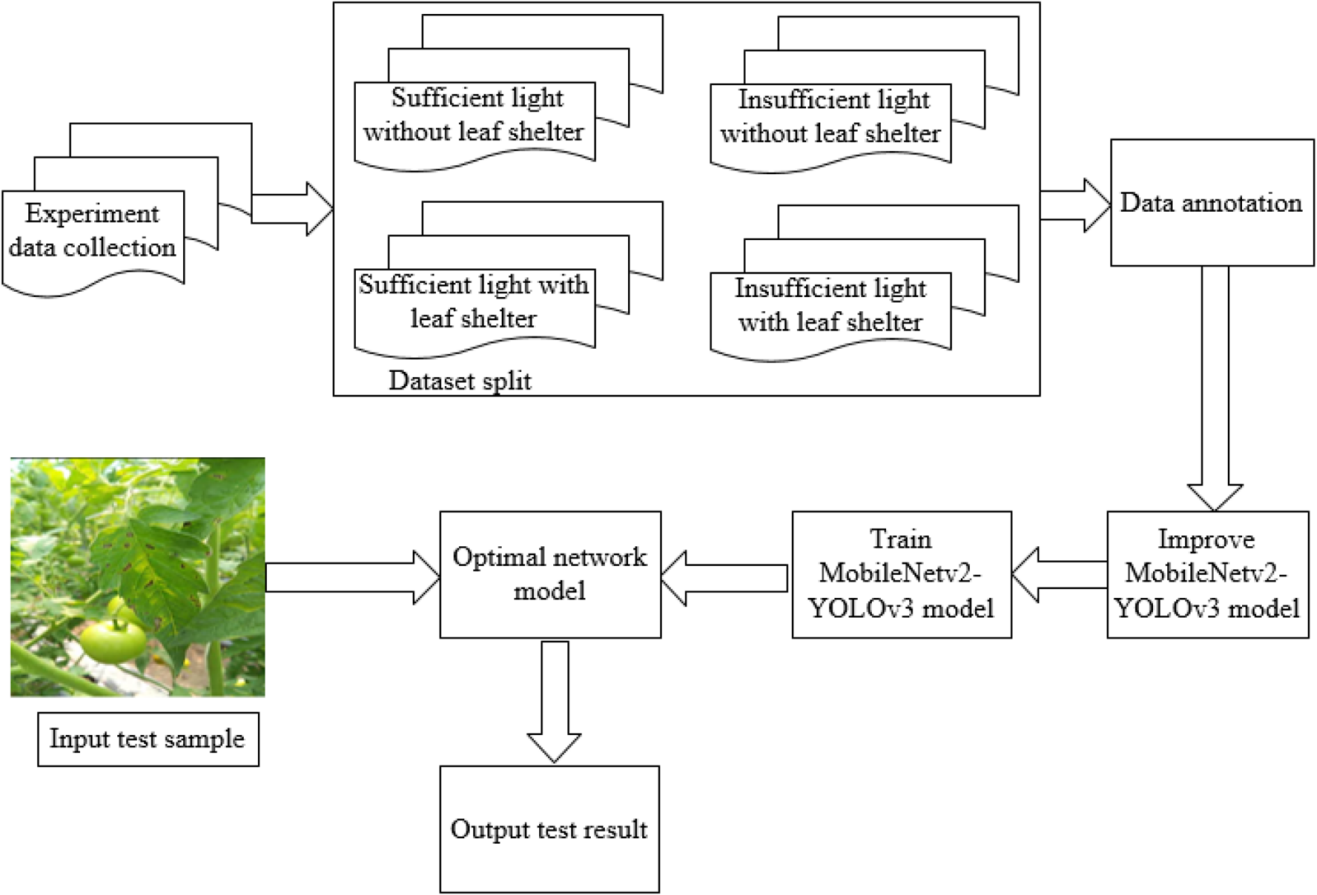
Flowchart of tomato disease detection network training

The training process of tomato gray leaf spot detection is shown in Fig. 3. This study adopts the method of comparative experiment and uses network model Faster-RCNN, SSD, YOLOv3 and MobileNetv2-YOLOv3 to perform the comparative experiment and verify the model effect on different datasets. Firstly, the collected data are split, annotated and stored in the format of PASCAL VOC. Secondly, hybrid learning combined with transfer learning, transfer learning alone and not using the pre-training methods are used to train the network model. The model parameters are corrected using the back-propagation algorithm to gradually reduce the loss function. The training process is completed when the average loss is less than 0.01 and the loss function is no longer reduced after multiple iterations.

The super parameter of the model is set to 32 sample number of each batch, the momentum factor is 0.9 and the initial learning rate is 0.001. In every 5000 iterations of training, the learning rate is reduced by 10 times, and the weight of the model is saved every 100 times of training.

## Results and discussion

### Model testing

**Fig. 4:**
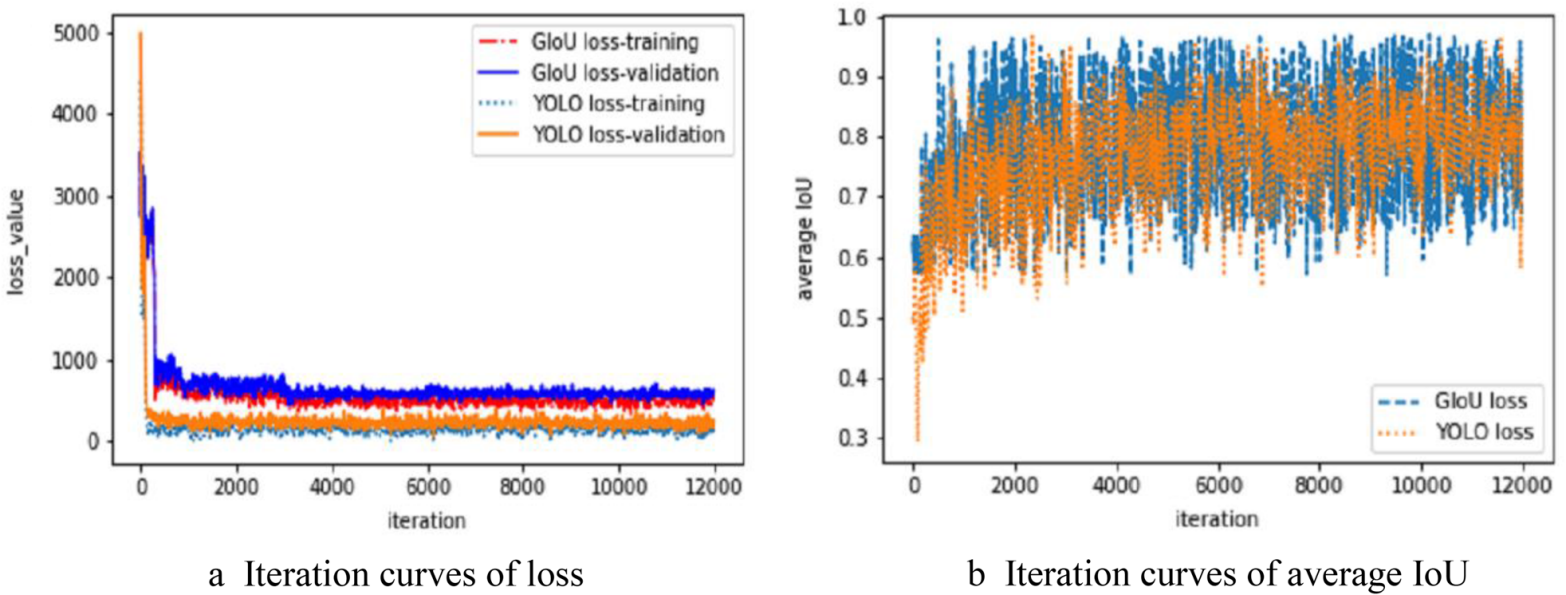
Iteration curves. **a** Iteration curves of loss, **b** Iteration curves of average IoU

**Fig. 5:**
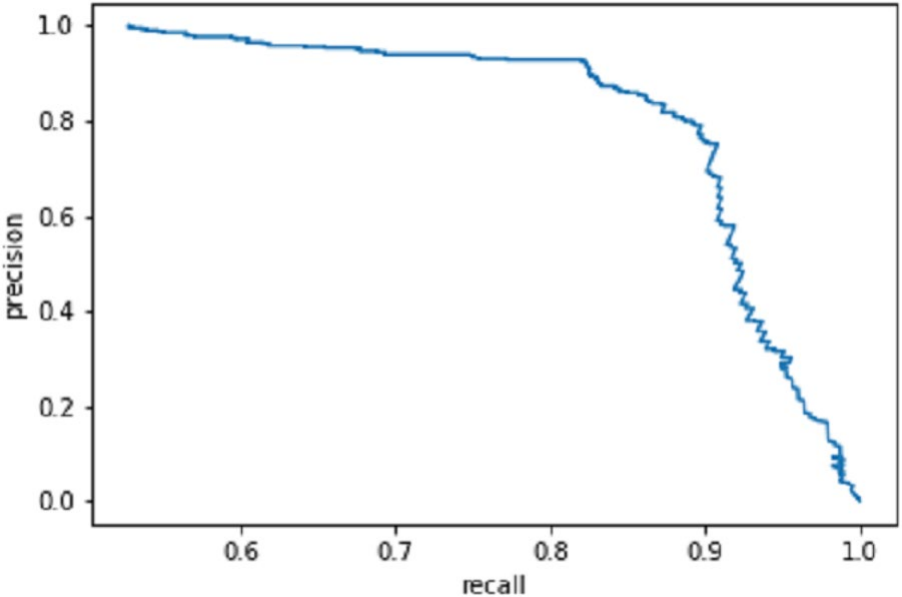
P–R curve

The GIoU loss function and the original YOLO loss function are used to train the MobileNetv2-YOLOv3 network model, and their training times are 10.6 and 12.8 h. The loss curve is shown in Fig. 5a. The loss value in the figure is the value of the loss function. The loss curve iterated on the training dataset using the model of YOLO loss is shown in the curve YOLO loss-train of Fig. 5a, and the loss curve iterated on the verification dataset is shown in the curve YOLO loss-val of Fig. 5a. The loss curve iterated by the model using the GIoU loss function on the training dataset is shown in the curve GIoU loss-train of Fig. 5a, and the loss curve iterated on the verification dataset is shown in the curve GIoU loss-val of Fig. 4a. The graph of average IoU for training is shown in Fig. 4b.

As shown in Fig. 4, the fitting degree of the model using GIoU Loss training is better than that of traditional YOLO loss on the verification dataset, and the average IoU value is significantly higher than that of YOLO loss. The model using YOLO loss is gradually stable after 3000 times, whilst the model using GIoU loss is gradually stable after 9000 times. The validation dataset is used to verify the advantages and disadvantages of the model, and the super parameters of the model are adjusted by comparing the loss curves of the validation and training datasets. Figure 4a shows the loss curve of the super optimal parameter iterated during the constant tuning process. The weights are saved 100 times per iteration, and the trained model is tested and evaluated. This study uses objective evaluation criteria (F1 score, AP value and average IoU value) to evaluate the advantages and disadvantages of the model through the weight saved for every 100 training sessions.

### Test results using mixup training and transfer learning

MobileNetv2-YOLOv3 network is used as the basic network. The traditional method does not use the pre-training model and trains all of the parameters with the training set from scratch. The transfer learning method uses the pre-training model of COCO dataset to train part of the layer parameters of the model. This study proposes the use of the pre-training model of mixup training combined with transfer learning to fine-tune the model. The testing results of the three kinds of methods in the test dataset are shown in Table 3. Compared with the traditional method, transfer learning significantly affects the improvement of the model. When mixup training and transfer learning are combined, the F1 score of the model increases by 3.73%, the AP value increases by 2.64% and the Average IoU value increases by 2.64%.

**Table 3:** Comparison of detection results using different training methods

Training methods	F1 score/ %	Average precision/ %	Average IoU/ %
Original method	88.99	87.65	80.49
Transfer learning method	91.53	89.38	82.57
Mixup + Transfer learning method	92.72	90.29	83.13

### Comparison of detection results of different backbone networks

This study compares different network models and backbone networks to prove the advantages of MobileNetv2-YOLOv3, and the test results in the test dataset are shown in Table 4. The network structure of YOLOv3 is more sophisticated than that of YOLOv2. Thus, the detection speed of the former is slightly lower than that of the latter, but the F1 score is increased by 6.20% and the AP value is increased by 6.24%. The recognition accuracy increases significantly. Table 3 indicates that the detection speed and weight size of YOLOv3-Tiny increase greatly, but the detection precision decreases obviously. By using MobileNetv1 as the backbone network of YOLOv3, the detection speed reaches 270 frames per second, and the weight size is only 23 MB. However, the AP value is decreased compared with that of the original YOLOv3. By using MobileNetv2 as the backbone network of YOLOv3 (the proposed MobileNetv2-YOLOv3), the F1 score and the AP value achieve the best results. Compared with the original YOLOv3, the AP value is increased by 1.77%, the F1 score is increased by 0.95%, the weight size is only 28 MB and the detection speed reaches 22 frames per second. Therefore, MobileNetv2-YOLOv3 network has obvious advantages because embedded terminal or mobile device is mostly used in image recognition of tomato leaf spot.

**Table 4:** Comparison of detection results using different backbone networks

Network models	Backbone networks	F1 score/ %	Average precision/ %	Weight size	Detection speed
YOLOv2	DarkNet-19	85.67	82.28	195 MB	70
YOLOv3	DarkNet-53	91.77	88.52	236 MB	62
YOLOv3-Tiny	Tiny	78.67	77.21	34 MB	220
YOLOv3	MobileNetv1	88.37	86.49	23 MB	270
YOLOv3	MobileNetv2	92.72	90.29	28 MB	246

### Test results using GIoU loss function

MobileNetv2-YOLOv3 is used as the basic network and the GIoU loss function is utilised to replace the original YOLOv3 loss function. After the training of the model, the test results are shown in Table 5. The F1 score is increased by 1.47%, the AP value is increased by 2.80% and the average IoU value is increased by 4.48%. Compared with the original YOLOv3, the test results are improved greatly. Therefore, the GIoU loss function greatly affects the accuracy of bounding box regression, which enables highly accurate detection of the location of tomato leaf spot disease.

**Table 5:** Detection results using GIoU loss function

Network models	F1 score/ %	Average precision/ %	Average IoU/ %
YOLOv3	91.77	88.52	82.49
MobileNetv2-YOLOv3	92.72	90.29	83.13
GIoU + MobileNetv2-YOLOv3	93.24	91.32	86.98

### Comparison of different backgrounds of disease

The different backgrounds of disease can affect the detection accuracy of the model greatly. Therefore, different backgrounds of the disease are taken as a control variable in this study, and the MobileNetv2-YOLOv3 model is used by the network model. Different backgrounds of test dataset are used to verify the test results, as shown in Table 6.

**Table 6:** Comparison of detection results under different backgrounds

Test set	F1 score/ %	Average precision/ %	Average IoU/ %
Sufficient light without leaf shelter	94.13	92.53	89.92
Sufficient light with leaf shelter	93.22	91.01	87.86
Insufficient light without leaf shelter	91.32	90.07	85.52
Insufficient light with leaf shelter	90.61	90.02	84.31

For the recognition of disease under the background of sufficient light without leaf shelter, the F1 score of the model can reach 94.13%, the AP value can reach 92.53% and the average IoU can reach 89.92%. Table 5 shows that the detection accuracy is slightly low for the recognition of disease under the background of insufficient light with leaf shelter. The reason is that the backgrounds have elements that mimic certain disease characteristics considering the actual application scenario. Thus, the network may learn them, which influences the recognition effect. The P–R curve of the whole test set is shown in Fig. 5.

### Comparison of different detection methods

**Fig. 6:**
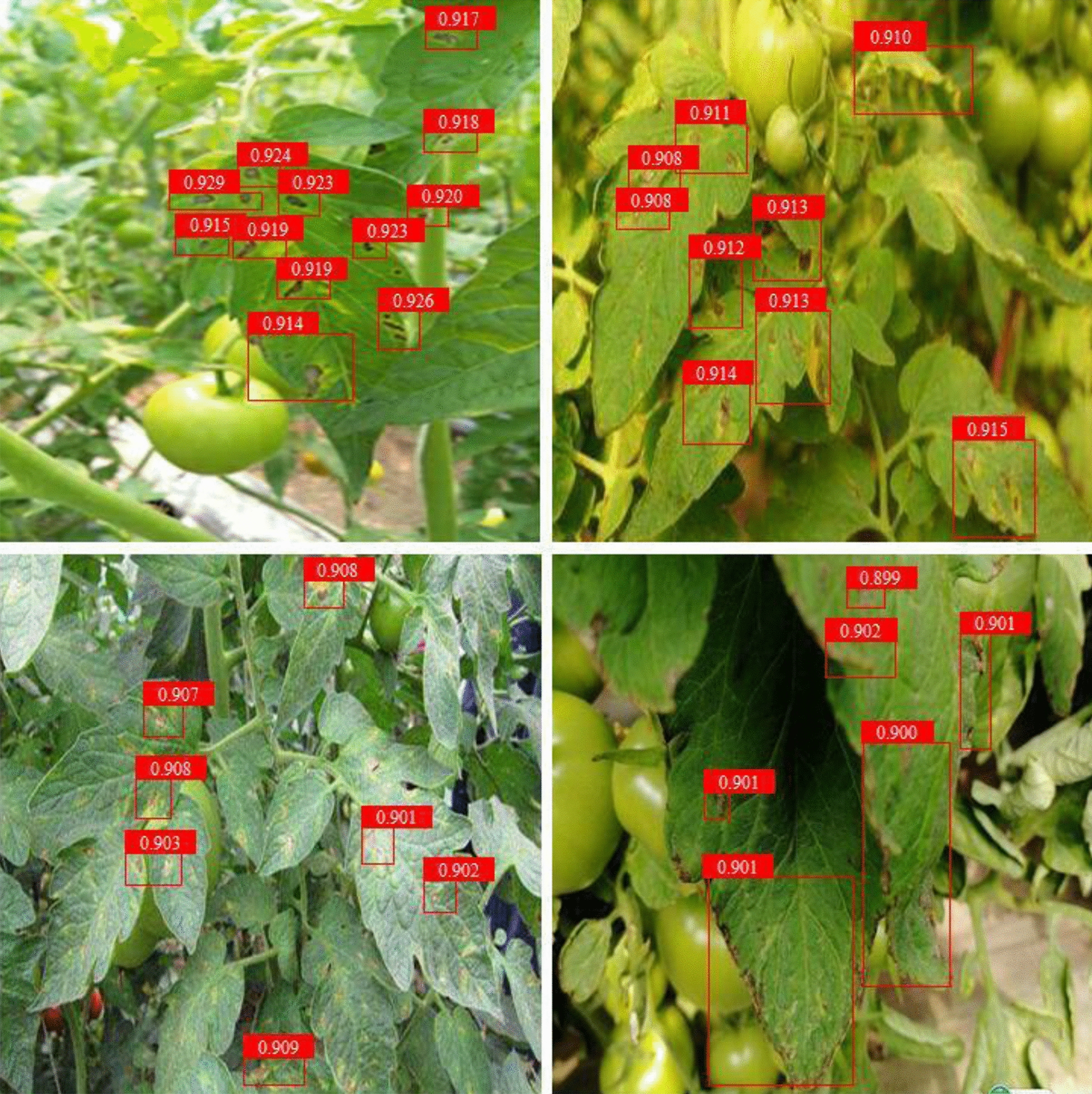
Effect diagram of the proposed detection method

Faster-RCNN, SSD and MobileNetv2-YOLOv3 are trained, and the test results are compared in different test sets (Table 7). Compared with SSD and Faster-RCNN, MobileNetv2-YOLOv3 significantly improves in accuracy, and the F1 score can reach more than 90% in the case of different backgrounds. For the detection of the disease under the background of sufficient light without leaf shelter, the F1 score and the AP value are 2.12 and 3.35% higher than those of SSD and 1.68% and 3.11% higher than those of Faster-RCNN. Figure 6 shows that MobileNetv2-YOLOv3 can miss the detection of tomato gray leaf spot under the condition of insufficient light (cloudy days) and leaf occlusion, and this performance is due to the effect on the detection accuracy of tomato gray leaf spot object under a darker background. Table 6 indicates that SSD is close to Faster-RCNN in terms of detection accuracy.

**Table 7:** Comparison of detection results using different network models

Test set	Network models	F1 score/ %	Average precision/ %
Sufficient light without leaf shelter	GIoU + MobileNetv2-YOLOv3	94.13	92.53
	SSD	92.01	89.18
	Faster-RCNN	92.45	89.42
Sufficient light with leaf shelter	GIoU + MobileNetv2-YOLOv3	93.22	91.01
	SSD	91.44	88.52
	Faster-RCNN	92.12	92.13
Insufficient light without leaf shelter	GIoU + MobileNetv2-YOLOv3	91.32	90.07
	SSD	89.67	87.96
	Faster-RCNN	90.01	88.33
Insufficient light with leaf shelter	GIoU + MobileNetv2-YOLOv3	90.61	90.02
	SSD	88.55	86.52
	Faster-RCNN	89.77	87.61

In terms of training time, the batch size of each iteration of the model is set to 32. The training times of Faster-RCNN and SSD are 16.7 and 12.2 h, respectively, whilst the training time of the proposed MobileNetv2-YOLOv3 is only 10.6 h. The full connection layer is removed from YOLOv3 and SSD. Thus, the training time will be significantly improved compared with that of Faster-RCNN. This study also adopts the method of mixup training and transfer learning to effectively reduce the training time. In terms of detection speed, MobileNetv2-YOLOv3 can achieve a detection speed of 246 frames/s, which is nearly 4 times faster than that of SSD and nearly 20 times faster than that of Faster-RCNN. The results of the above-mentioned five groups of comparative tests indicate that the proposed MobileNetv2-YOLOv3 lightweight neural network can effectively identify tomato gray leaf spot region under natural environment. The recognition accuracy and speed of the proposed method have significant advantages over other methods.

## Conclusions and future directions

### Conclusions

1. An improved recognition method of tomato gray leaf spot based on MobileNetv2-YOLOv3 lightweight neural network is proposed. The test results show that, in the test dataset of images captured under the background of sufficient light without leaf shelter in natural environment, the F1 score and AP value are 94.13% and 92.53%, and the average IOU value is 89.92%. In all of the test datasets, the F1 score and AP value of model detection reach 93.24 and 91.32%, respectively. The loss function of GIoU regression box is used to replace the MSE mean square error part of the traditional loss function border regression, and the average IoU is as high as 86.98%, which provides good technical support for tomato gray leaf spot localisation.
2. A lightweight neural network model is proposed by improving the model. The model occupies 28 MB of memory. For an image of 416 × 416, the detection speed can reach 16.9 ms on GPU and 80.9 ms on CPU, which can be used to transplant to embedded and mobile terminal. A method combining mixup training and transfer learning is proposed to transfer the knowledge learned from the COCO dataset to the tomato gray leaf spot recognition model, which improves the generalisation ability of the model and greatly reduces the training time and resources.
3. The recognition accuracy of Faster-RCNN and SSD in different scenarios is compared with those of different models in terms of the detection accuracy and calculation speed to verify the feasibility and superiority of the proposed method. In natural environment, the F1 score and AP value are 4.06% and 3.61% higher than those of SSD and 3.92% and 3.38% higher than those of Faster-RCNN. MobileNetv2-YOLOv3 can achieve a detection speed of 246 frames/s, which is nearly 4 times faster than that of SSD and nearly 20 times faster than that of Faster-RCNN. The proposed method has significant advantages over other methods.

### Future directions

Tomato is a widely planted crop in the world with abundant nutrients. In this study, MobileNetv2-YOLOv3 is applied to the early detection of tomato gray leaf spot disease to achieve non-destructive detection. However, some problems still need to be solved urgently.

1. This work only aims to detect tomato gray leaf spot disease. Other kinds of common diseases exist in tomato. Thus, the research of disease types on this basis should be increased to realise the detection of other kinds of diseases.
2. Given the achievements obtained in this study through the combination of software and hardware, the proposed algorithm should be run on a computer platform or a mobile app to enable application to actual production for facilitating farmers’ access to aid for their crops anytime and anywhere.
3. This study realises the early detection of tomato gray leaf spot disease, which can play a role in timely detection. Subsequent studies will acquire temperature and humidity information, pathogenic spore information, soil information and environmental information through multiple sensors, fuse multisource data and construct an early warning model of tomato gray leaf spot disease based on multidata fusion to further realise early warning when the disease does not occur.

## Data Availability

Given that the data used in this study were acquired by self-collection, the dataset is being further improved. Thus, the dataset is unavailable for the time being.

## References

[1] Mariko T, Hiroshi E (2015). How and why does tomato accumulate a large amount of GABA in the fruit?. Front Plant Sci.

[2] Fuentes A, Yoon S, Youngki H, Lee Y, Park DS (2016). Characteristics of tomato plant diseases—a study for tomato plant disease identification. Proc Int Symp Inf Technol Converg.

[3] Barbedo JGA (2016). A review on the main challenges in automatic plant disease identification based on visible range images. Biosys Eng.

[4] Krizhenvshky A, Sutskever I, Hinton G. Imagenet classification with deep convolutional networks. In Proceedings of the Conference Neural Information Processing Systems (NIPS), Lake Tahoe, NV, USA, 3–8 December 2012; p 1097–1105.

[5] Lin M, Chen Q, Yan S. Network in Network. arXiv 2013, arXiv:arXiv:1312.4400.

[6] Simonyan K, Zisserman A. Very deep convolutional networks for large-scale image recognition. arXiv 2014, arXiv:arXiv:1409.1556.

[7] Szegedy C, Liu W, Jia Y, Sermanet P, Reed S, Anguelov D, Erhan D, Vanhoucke V, Rabinovich A. Going deeper with convolutions. In Proceedings of the 2015 IEEE Conference on Computer Vision and Pattern Recognition, Boston, MA, USA, 7–12 June 2015; pp. 1–9.

[8] He K, Zhang X, Ren S, Sun J. Deep residual learning for image recognition. In Proceedings of the 2016 IEEE Conference on Computer, Vision, Pattern Recognition, Las Vegas, NV, USA, 27–30 June 2016; pp. 770–778.

[9] He K, Zhang X, Ren S, Sun J. Identity Mapping in deep residual networks. arXiv 2016, arXiv:arXiv:1603.05027.

[10] Xie S, Girshick R, Dollár P, Tu Z, He K. Aggregated residual transformations for deep neural networks. arXiv 2017, arXiv:arXiv:1611.05431.

[11] Girshick R, Donahue J, Darrell T, et al. Rich feature hierarchies for accurate object detection and semantic segmentation[C]//IEEE conference on Computer Vision and Pattern Recognition. Columbus: IEEE, 2014: 580-587.

[12] He K, Zhang X, Ren S, Sun J. Spatial pyramid pooling in deep convolutional networks for visual recognition. In Proceedings of the 2014 IEEE International Conference of European Conference on Computer Vision, Zurich, Switzerland, 6–12 September 2014; pp. 346–361.

[13] Ren S, He K, Girshick R, Sun J (2016). Faster R-CNN: towards real-time object detection with region proposal networks. IEEE Trans Pattern Anal Mach Intell.

[14] Liu W, Anguelov D, Erhan D, Szegedy C, Reed S, Fu C, Berg AC. SSD: Single Shot MultiBox Detector. In Proceedings of the European Conference on Computer Vision—ECCV, Amsterdam, The Netherlands, 8–16 October 2016; pp. 21–37.

[15] Redmon J, Divvala S, Girshick R, Farhadi A. You only look once: Unified, Real-Time Object Detection. In Proceedings of the IEEE Conference on Computer Vision and Pattern Recognition, Las Vegas, NV, USA, 26 June–1 July 2016; pp. 779–788.

[16] Howard AG, Zhu M, Chen B, Kalenichenko D, Wang W, Weyand T, Andreetto M, Adam H. MobileNets: efficient convolutional neural networks for mobile vision applications. arXiv, 2017; arXiv:1704.04861.

[17] Iandola FN, Han S, Moskewicz MW, Ashraf K, Dally WJ, Keutzer K. SqueezeNet: AlexNet-level accuracy with 50 × fewer parameters and < 0.5 MB model size. arXiv, 2016; arXiv:1602.07360.

[18] Mohanty SP, Hughes DP, Salathé M (2016). Using deep learning for image-based plant disease detection. Front Plant Sci.

[19] Amara J, Bouaziz B, Algergawy A. A deep learning based approach for banana leaf diseases classification[M]//MITSCHANG B. Lecture Notes in Informatics (LNI). Bonn: Gesellschaft Für Informatik, 2017:79-88.

[20] Wang G, Sun Y, Wang J (2017). Automatic image-based plant disease severity estimation using deep learning. Comput Intell Neurosci.

[21] Ferentinos KP (2018). Deep learning models for plant disease detection and diagnosis. Comput Electron Agric.

[22] Barbedo Arnal, Garcia Jayme (2019). Plant disease identification from individual lesions and spots using deep learning. Biosys Eng.

[23] Lee SH, Chan CS, Wilkin P, et al. Deep-plant: Plant identification with convolutional neural networks. IEEE International Conference on Image Processing. IEEE,2015:452-456.

[24] Pan SJ, Yang QA survey on transfer learning. IEEE Transactions on Knowledge & Data Engineering, 2010,22(10):1345-1359.

[25] Srdjan S, Marko A, Andras A (2016). Deep neural networks based recognition of plant diseases by leaf image classification. Comput Intell Neurosci.

[26] Liu B, Zhang Y, He DJ (2017). Identification of apple leaf diseases based on deep convolutional neural networks. Symmetry.

[27] Too EC, Li Y, Njuki S (2018). A comparative study of fine-tuning deep learning models for plant disease identification. Comput Electron Agric.

[28] Picon A, Alvarez-Gila A, Seitz M, Ortiz-Barredo A, Echazarra J, Johannes A (2019). Deep convolutional neural networks for mobile capture device-based crop disease classification in the wild. Comput Electron Agric.

[29] Selvaraj MG, Vergara A, Ruiz H (2019). AI-powered banana diseases and pest detection. Plant Methods.

[30] Zhong Yong, Zhao Ming (2020). Research on deep learning in apple leaf disease recognition. Comput Electron Agric.

[31] Durmus H, Gunes E.O, Kirci M, Disease detection on the leaves of the tomato plants by using deep learning. In Proceedings of the 6th International Conference on Agro-Geoinformatics, Agro-Geoinformatics 2017, Fairfax, VA, USA, 7–10 August 2017.

[32] Brahimi M, Arsenovic M, Laraba S, Sladojevic S, Boukhalfa K, Moussaoui A. Deep learning for plant diseases: detection and saliency map visualisation. In Human and Machine Learning; Zhou J, Chen F, Eds.; Springer International Publishing: Cham, Switzerland, 2018; pp. 93–117. ISBN 978-3-319-90402-3.

[33] Aravind KR, Raja P, Aniirudh R (2018). Tomato crop disease classification using pre-trained deep learning algorithm. Procedia Comput Sci.

[34] Karthik R, Hariharan M, Anand Sundar, Mathikshara Priyanka, Johnson Annie, Menaka R (2020). Attention embedded residual CNN for disease detection in tomato leaves. Applied Soft Comput.

[35] Fuentes A, Yoon S, Kim SC, Park DS (2022). A robust deep-learning-based detector for real-time tomato plant diseases and pests recognition. Sensors.

[36] Fuentes AF, Yoon S, Lee J, Park DS (2018). High-performance deep neural network-based tomato plant diseases and pests diagnosis system with refinement filter bank. Front Plant Sci.

[37] Fuentes AF, Yoon S, Park DS (2019). Deep learning-based phenotyping system with glocal description of plant anomalies and symptoms. Front Plant Sci.

[38] Zhang Y, Song C, Zhang D (2020). Deep learning-based object detection improvement for tomato disease. IEEE Access.

[39] Barbedo JG (2018). Factors influencing the use of deep learning for plant disease recognition. Biosyst Eng.

[40] Redmon J, Farhadi A. YOLOv3: An incremental improvement. arXiv 2018, arXiv:1804.02767 [cs], 1–6.

[41] Redmon J, Farhadi A. YOLO9000: Better, Faster, Stronger. 2016

[42] He KM, Zhang XY, Ren SQ et al. Deep residual learning for image recognition [C]//2016 IEEE Conference on Computer Vision and Pattern Recognition (CVPR), June 27-30, 2016, Las Vegas, NV, USA. New York: IEEE, 2016:770-778.

[43] Ma J, Shao W, Ye H (2018). Arbitrary-oriented scene text detection via rotation proposals. IEEE Trans Multimedia.

[44] Sandler M, Howard A, Zhu M, et al. Mobilenetv2: Inverted residuals and linear bottlenecks. IEEE Conference on Computer Vision and Pattern Recognition. Salt Lake City: IEEE, 2018: 4510-4520.

[45] Huang G, Liu Z, Van Der Maaten L, Weinberger KQ. Densely connected convolutional networks. In Proceedings of the IEEE Conference on Computer Vision and Pattern Recognition, Honolulu, HI, USA, 21–26 July 2017; pp. 4700–4708

[46] Yosinski J, Clune J, Bengio Y, et al. How transferable are features in deep neural networks?[C]//Advances in Neural Information Processing Systems, 2014: 3320-3328.

