# Correction to: Early recognition of tomato gray leaf spot disease based on MobileNetv2-YOLOv3 model

**DOI:** 10.1186/s13007-021-00708-7

**Published:** 2021-02-09

**Authors:** Jun Liu, Xuewei Wang

**Affiliations:** Facility Horticulture Laboratory of Universities in Shandong, Weifang University of Science and Technology, Weifang, 262700 Shandong China

## Correction to: Plant Methods (2020) 16:83 10.1186/s13007-020-00624-2

Unfortunately, in the original publication of the article [1], the symbol (+) was incorrectly mentioned as (=) in Equation 1.

The corrected Eq. 1 is given below:

1 $$ \begin{aligned} Loss & = Loss_{coord} + Loss_{obj} + Loss_{class} \\ & = \lambda_{coord} \sum\limits_{i = 0}^{{s^{2} }} {\sum\limits_{j = 0}^{B} {l_{ij}^{obj} } } \left[ {\left( {x_{i} - \hat{x}_{i} } \right)^{2} + \left( {y_{i} - \hat{y}_{i} } \right)^{2} } \right] \\ & \quad + \lambda_{coord} \sum\limits_{i = 0}^{{s^{2} }} {\sum\limits_{j = 0}^{B} {l_{ij}^{obj} } } \left[ {\left( {\sqrt {w_{i} } - \sqrt {\hat{w}_{i} } } \right)^{2} + \left( {\sqrt {h_{i} } - \sqrt {\hat{h}_{i} } } \right)^{2} } \right] \\ & \quad + \sum\limits_{i = 0}^{{s^{2} }} {\sum\limits_{j = 0}^{B} {l_{ij}^{obj} } } \left( {C_{i} - \hat{C}_{i} } \right)^{2} + \lambda_{noobj}  \sum\limits_{i = 0}^{{s^{2} }} {\sum\limits_{j = 0}^{B} {l_{ij}^{obj} } } \left( {C_{i} - \hat{C}_{i} } \right)^{2} \\ & \quad + \sum\limits_{i = 0}^{{s^{2} }} {l_{ij}^{obj} } \sum\limits_{c \in class} {\left( {p_{i} (c) - \mathop {\hat{p}}\limits_{i} (c)} \right)}^{2} \\ & \quad \\ \end{aligned} $$

The original article has been corrected.
